# Invader soil conditioning impacts invader and native plant performance

**DOI:** 10.1093/aobpla/plag005

**Published:** 2026-02-03

**Authors:** Stuart T Schwab, Bea Portez, George Darrel Jenerette, Loralee Larios

**Affiliations:** Botany and Plant Science Department, University of California Riverside, 2142 Batchelor Hall, Riverside, CA 92521, United States; Botany and Plant Science Department, University of California Riverside, 2142 Batchelor Hall, Riverside, CA 92521, United States; Botany and Plant Science Department, University of California Riverside, 2142 Batchelor Hall, Riverside, CA 92521, United States; Conservation Biology Program, University of California Riverside, 2118 Batchelor Hall, Riverside, CA 92521, United States; Botany and Plant Science Department, University of California Riverside, 2142 Batchelor Hall, Riverside, CA 92521, United States; Conservation Biology Program, University of California Riverside, 2118 Batchelor Hall, Riverside, CA 92521, United States

**Keywords:** greenhouse experiment, functional trait, plant-soil-biota-interaction, tradeoff

## Abstract

Plant invaders can promote invasion success through interactions with soil-biota (i.e. soil-conditioning), forming feedback, which can change in strength and direction over time. Thus, native plant responses to invader soil-conditioning dynamics may be dependent on the degree of invasion and could additionally be mediated by plant functional traits. To investigate the temporal dynamics of invader-soil-conditioning and the role of traits in mediating plant responses, we conducted a greenhouse experiment focusing on *Oncosiphon pilulifer*, an invasive annual forb spreading across the Southwestern United States and Western Australia. We grew *Oncosiphon* and six native plants in live whole soil vs sterilized whole soil inocula from an existing *Oncosiphon* invasion gradient, with four levels of invasion ranging from uninvaded, small patches, large monocultures, and the origin point of invasion resulting in a space-for-time substitution. We measured plant biomass, mycorrhizal root colonization, and leaf and root traits. We found native plant growth was reduced with soil from patchily invaded soils, while mycorrhizal root colonization rates were reduced with *Oncosiphon* monoculture soil. *Oncosiphon* itself experienced reduced growth over the course of invasion, with consistently low root colonization. Our trait analysis suggests that an interaction between root and leaf traits can mediate plant vulnerability to invader impacts on soil-biota.

## Teaser

Invasive plants often enhance invasion success by altering soil communities in ways that inhibit native species. We studied soil interactions associated with the emerging invader Oncosiphon pilulifer and found that it suppresses native plant growth while also limiting its own performance over time. We further show that plant traits, such as leaf and root thickness, influence how strongly species respond to these soil-mediated effects, highlighting trait-based sensitivity to invasion.

## Introduction

Plant interactions with soil-biota can drive soil conditioning through root exudates and litter (Hu et al. 2018, Yates et al. 2024), which form feedback that influences plant abundance (Klironomos 2002), and competitive outcomes in uninvaded communities (Kandlikar et al. 2021). Soil conditioning by plants can also contribute to invasion success if the plant invader’s feedback exacerbates existing competitive differences between native and invasive plants (Reinhart and Callaway 2006). Plant invaders commonly interact with beneficial soil symbionts including arbuscular mycorrhizal fungi (AMF), which can promote invasion success by conditioning the soil for more of the same AMF species thereby increasing invader fitness in a positive feedback (i.e. enhanced mutualisms: Reinhart and Callaway 2006, Yu et al. 2022), or by conditioning soils to reduce AMF presence, which native plant species are differentially dependent on (i.e. degraded mutualisms: Vogelsang and Bever 2009, Grove et al. 2017). Importantly, feedback between plants and soil biota is dynamic and can shift over the course of invasion to promote or reduce growth of native and invasive species alike depending on how long the plant invader has conditioned the soil (Hawkes et al. 2013, Ke et al. 2021). For example, plant invasion can result in a transition from neutral to negative feedback in the invader via increases in soil pathogens, but this is expected to happen over longer time frames as pathogen populations may be initially naïve to the plant invader (Diez et al. 2010). Understanding how the feedback from invader soil conditioning changes over the course of invasion can inform when management can intervene to minimize potential soil-biota legacy effects.

Plant invaders alter plant–soil interactions through changes in belowground soil-biota (i.e. soil-biota-feedback), where plant density dependence is often assumed (Bever et al. 1997); however, plant invader abundance is not always directly linked to the degree of change in soil-biota (Chung 2023). For instance, in enhanced mutualism feedback (i.e. AMF increase invasive plant growth), an established invader might reduce their dependence on mycorrhizae if their dominance over time enables it to depend less on fungi (Seifert et al. 2009). Alternatively, plant invaders might develop stronger mycorrhizal relationships over time through increased probability of encountering a compatible symbiont, or by conditioning the soil community to increase the amount of their compatible symbionts (Reinhart and Callaway 2006). In contrast, in degraded mutualisms feedback (i.e. plant invader reduces AMF that native species depend on), allelopathic invaders can reduce AMF presence early in patchy stages of invasion by directly inhibiting AMF, or they can reduce AMF presence by excluding native plant hosts over long enough time that AMF populations deplete indirectly from a lack of native plant hosts (Grove et al. 2017). Not all native plants are impacted by losses in AMF caused by invader soil conditioning to the same degree, as some native plants have a more facultative relationship with mycorrhizae or are non-mycorrhizal (Bunn et al. 2015). Predicting the sensitivity of native plants to invader soil conditioning remains a challenge; however, integrating key functional traits that reflect fundamental tradeoffs in resource acquisition and growth strategies may help determine the sensitivity of native plants to changes in soil-biota (Laliberté 2017).

Plant functional traits may play an important role in mediating the sensitivity of native plants to invader soil conditioning, and could elucidate mechanisms of native resistance. While traits have been identified to mediate soil-biota-feedback for both native (Cortois et al. 2016, Xi et al. 2021) and invasive species (Dawson 2015), functional traits have yet to be linked with native responses to invader soil conditioning. Nevertheless, emerging trait frameworks highlight the importance of considering trade-offs in plant strategies. Differences in specific root length (SRL) may reflect a trade-off in resource acquisition strategies, resulting in a pattern of high SRL species exhibiting a ‘do it yourself’ soil exploration strategy and lower SRL species utilizing an ‘outsourcing’ strategy that relies on AMF partnerships to acquire belowground resources (McCormack and Iversen 2019, Bergmann et al. 2020). In another key plant strategy trade-off, plants may invest differentially in growth and defense, such that plants may exhibit a fast growth strategy that is less well defended against herbivores and pathogens or a slower growth strategy that is better defended against herbivores or pathogens (Weigelt et al. 2021, Xi et al. 2021). While individual traits have been linked to soil-biota-feedback in uninvaded systems, how traits relate to soil-biota-feedback in an invaded system, and how functional traits mediate native responses to invader soil conditioning remains unclear.

*Oncosiphon pilulifer* is a novel annual invasive forb in the Southwest United States and Western Australia, native to South Africa. Within California, *Oncosiphon’s* original introduction point is known (Lake Perris State Park, CA) and thought to have been originally introduced with a soil shipment from South Africa in the 1980’s. There are surrounding reserves that have experienced differing degrees of invasion from single-point introductions typically near visitor parking lots. *Oncosiphon* has substantially altered the landscapes within reserves and is expanding in density and cover regionally across Southern California, Arizona, and Western Australia (Hedrick and McDonald 2020, Schwab et al. 2023). As a recently expanding invader, less is known about the potential for *Oncosiphon*-driven changes in soil-biota (i.e. soil conditioning) to restructure plant and soil community dynamics. Novel invasions also provide unique opportunities to elucidate the temporal development of invader soil conditioning and associated impacts on native plants (Teste et al. 2019). In addition to differences in invasion levels among reserves, there are areas within each reserve that differ in invader abundance, providing an opportunity to explore the temporal development of invader soil conditioning within and between sites.

To better identify how a new species in a community can influence soil-biota-feedback, we tested a series of hypotheses on the temporal development of *O. pilulifer* soil conditioning. Using this recent invasion as a model for the dynamics of invader soil conditioning we asked, how does *Oncosiphon* soil conditioning influence soil-biota-feedback for resident plant species and itself? Increases in *Oncosiphon* growth with soil-biota from more heavily invaded areas would indicate positive soil-biota-feedback. We would expect corresponding increases in AMF root colonization rates for *Oncosiphon* plants grown in inocula with higher *Oncosiphon* cover, which would indicate enhanced mutualisms soil-biota-feedback. Conversely, decreases in native plant growth associated with soil-biota from more heavily invaded soil and corresponding decreases in native AMF root colonization rates would indicate natives are experiencing degraded mutualisms soil-biota-feedback.

We further asked how plant functional traits shape native responses to invader soil conditioning, and how these trait-soil-biota relationships change over the course of invader soil conditioning? Plants with higher SLA may be more sensitive to belowground enemies due to growth-defense tradeoffs, while plants with higher SRL may be less sensitive to changes in mycorrhizal soil-biota as they may reflect a ‘do-it-yourself’ strategy rather than a mutualist dependent strategy. In the case of degraded mutualism feedback, the reduction in symbiont availability would have stronger effects on plants with high mycorrhizal dependency (i.e. species with low SRL). We predict that low SRL species may shift from a positive association with soil-biota to a neutral or negative association as AMF presence is reduced with increasing invader soil conditioning. High SRL species should have a consistently neutral or slightly negative association with the degree of invader soil conditioning in degraded mutualisms soil-biota-feedback. Enhancing our understanding of how invader soil conditioning develops over time to influence both the invader and native plant performance, and how the functional traits of native plants may mediate their sensitivity to invader soil conditioning can elucidate mechanisms of invasion success and native resistance to invasion.

## Materials and methods

### Soil inocula collection

To assess the development of *Oncosiphon*’s soil conditioning, we collected soil cores from three reserves and four invasion levels within each reserve in April 2019. We collected soil from Lake Perris State Recreation Area (33.868530, −117.176620), Motte Rimrock Reserve (33.800570, −117.255322), and Lake Matthew’s Estelle Mountain Preserve (33.808138, −117.426358). To capture a gradient of soil conditioning, we used a quasi-space-for-time substitution developed after several preliminary scouting trips to identify within season invasion patterns. *Oncosiphon* has many stems, making individual identification difficult; however, we found a consistent pattern of invasion where small (<0.25 m^2^) patches form before large (>20 m^2^) monocultures form. Thus, within each reserve, invasion levels were determined as ‘uninvaded’ having no *Oncosiphon* present for at least 50 m in every direction, ‘patchy’ as *Oncosiphon* stands under 0.25 m^2^ with at least 1 m with no *Oncosiphon* between patches, ‘monoculture’ having 99% *Oncosiphon* cover over at least 20 m^2^, and ‘origin’ soils were determined as the original sightings of *Oncosiphon* within the reserve. These designations broadly follow the spatial pattern of invasion proposed by Peters et al. (2004), where initial invasions are characterized by localized populations and spread until patches are connected. Additionally, the degree of invasion within each site was discussed with management practitioners, and at the time of collection *Oncosiphon* was a novel invasion, so the densities generally correspond with the local manager’s knowledge of invasion history. We included origin soils to estimate the potential for long-term soil conditioning, as negative feedback develops over long time scales (Diez et al. 2010), and impacts on AMF may also be dynamic over longer time periods (Reinhart and Callaway 2006, Seifert et al. 2009).

We collected three soil cores 10 cm diameter × 15 cm deep for each soil conditioning level within a site (i.e. 3 samples × 4 soil histories ∼12 soil samples per site) for a total of 36 live experimental soil cores. We pooled three cores per invasion soil conditioning level (i.e. 3 soil cores pooled to 1 for sterilized comparison) for each site and used them as a sterilized biological control soil (36 total cores pooled to 12 inoculants; see Brinkman et al. 2010 for statistical and methodological explanations and justifications for this decision), making a total of 48 inocula (36 experimental cores + 12 sterilized controls for each soil type = 48). See Supplementary Appendix a for full details on soil handling techniques and preparation of inocula.

### Greenhouse experiment

To assess soil-biota-feedback within the *Oncosiphon* invasion gradient, we grew *Oncosiphon* and six annual native plants in a greenhouse experiment. The species include *Amsinckia intermedia* (Boraginaceae), *Eschscholzia californica* (Papaveraceae), *Lasthenia californica* (Asteraceae), *Layia platyglossa* (Asteraceae), *Lupinus bicolor* (Fabaceae), and *Nemophila menziesii* (Boraginaceae). These species represent a breadth of common and rare native plants with different resource acquisition strategies. All species occur at all reserves, but at differing frequencies and abundances. Seeds were purchased from S&S Seeds (Carpinteria, CA), and each sample was grown individually in a 300 ml pot. To isolate the contribution of soil-biota to plant growth, we added a small amount of live soil inocula to sterilized bulk soil at a rate of 1:30 (i.e. 3.34% of soil by volume). By including a small amount of soil for the inoculations, our design effectively mitigates the influence of nutrient dynamics during plant invasion (Brinkman et al. 2010). The majority of the pots (i.e. 96.66% by volume) were filled with sterilized bulk soil that was collected from each reserve, sieved with a 2 mm soil sieve, then mixed with a University of California sand mix (see Supplementary Table S1).

In the greenhouse, we grew three individuals for each species per inoculum. Therefore, for each soil history, we had 189 samples for live inocula (3 sites × 3 soil cores × 7 species × 3 plant replicates) and 63 for sterile (3 sites × 7 species × 3 plant replicates) resulting in a total of 1008 individuals (252 samples × 4 soil histories = 1008). We implemented a completely random design and further randomized tray locations within the greenhouse weekly during the experiment. Plants were grown over 10 weeks in a University of California Riverside greenhouse and watered with spray emitters for 10 minutes three times a week for the first 7 weeks, then additional watering *ad libitum* for the remaining 3 weeks ranging from an additional 5 minutes at 2 pm on non-watering days, to 5 minutes at 2 pm every day depending on plant wilting due to increasing temperatures.

After 10 weeks we harvested plants to assess plant growth, plant functional traits, and percent root mycorrhizal colonization. We calculated the contributions of soil-biota as a ratio to estimate soil-biota-feedback: $ln(\frac{dry\;shoot\;mass\;live}{\begin{array}{c} average\;sterile\;dry\;shoot\;mass\;per\;\\ soil\;conditioning\;level\;within\;reserve \end{array}})$. In this design, positive values indicate greater growth in live soil (i.e. positive soil-biota-feedback from mutualists), and negative values indicate decreased growth in live soil (i.e. negative soil-biota-feedback from belowground enemies) (Brinkman et al. 2010). For these annual plants, root biomass was highly correlated with root length as we calculated root length based on the whole root system (Pearson’s product moment correlation *R*^2^ = 0.72, *P* < 0.0001) to minimize the potential correlation in SRL with our feedback response variable, we focus on just shoot biomass. Moreover, the shoot masses were tightly correlated with full plant mass (Pearson’s product moment correlation *R*^2^ = 0.95, *P* < 0.0001).

During harvest, plants were first cut at the base of the shoot and had the first fully formed true leaf removed with a razorblade. We then scanned the leaf and later assessed leaf area with imageJ (Schneider et al. 2012). Shoot dry mass and leaf dry mass were taken after shoot and leaf samples were placed in a 60°C drying oven for 48 hours. To acquire SRL and root mycorrhizal colonization, plants were de-potted and roots were washed and cleaned free of all soil. We scanned the full root system (LA2400 Regent Instruments Scanner) in a thin film of water within 24 hours of harvest, and the full length of the entire root system was measured with ‘WinRhizo^TM^’ software. Oven-dried roots become brittle that makes AMF colonization estimates difficult and less accurate. Therefore, to obtain both root dry mass for SRL estimates and mycorrhizal colonization we air dried samples for 2 weeks. We then measured the full root system air-dried mass, and subsampled a small portion of fine roots ensuring we had at least 10 independent roots at least 1 cm long for mycorrhizal analyses (per Brundrett et al. 1996), then re-measured the remaining roots to acquire the percentage of oven dried roots by mass (i.e.$\frac{air\;dry\;root\;mass-subsample}{total\;air\;dried\;root\;mass}$). The remaining roots were placed in a 60°C drying oven for 48 hours, and the oven dried air mass of the remaining sample was measured. To estimate the root dry mass of the samples, we calculated dry mass as the oven dry weight/percentage oven dried.

Specific leaf area (SLA) was calculated as leaf area (cm^2^) divided by leaf dry mass (g), and SRL was calculated as total root length (m)/root dry mass (g). Root mycorrhizal colonization was estimated after roots were cleared with 2.5% KOH and stained with 0.5% Trypan Blue. We utilized the point intersect method (Brundrett et al. 1996) using ten 1 cm roots per plant with 10 fields of view per root at 400× magnification.

We lost 120 plants due to mortality across all soil histories and species, except for *Amsinckia* (see Supplementary Figure S1 for distribution across species and soil histories; notably mortality was highest in *Lupinus* and *Layia*). In most instances, at least one plant replicate was present for a given soil inocula; however, in two instances we lost all plant replicates for a soil inocula. Specifically, all *Layia* individuals in the sterile uninvaded inoculum from Lake Perris State Park, as well as all *Lupinus* individuals in sterile origin inoculum from the Motte Rimrock reserve died during the experiment, so we took the average of each species in the corresponding inocula from the other reserves to calculate soil-biota-feedback. However, this statistical approach to estimating feedback is not strongly altered by pooling controls at this level of spatial resolution (Brinkman et al. 2010). Additionally, we lost 27 SLA values, due to incorrect imagery scans (7) or due to inaccuracy in mass estimates arising from leaf tissue sticking to the tape from leaf scans (20). Additionally, we lost 21 SRL values due to inaccurate estimates of total root mass arising from the subsampling for AMF colonization prior to calculating root biomass.

### Analysis

To evaluate *Oncosiphon’s* response to its own soil conditioning, we ran linear mixed effects models with either soil-biota-feedback or percent root mycorrhizal colonization as the response variable, with soil conditioning as the fixed effect and soil core nested in reserve as the random term. We similarly evaluated native responses to *Oncosiphon* soil conditioning, using soil-biota-feedback as the response with soil conditioning, species, and the interaction (i.e. soil conditioning × species) as the fixed effects with soil core nested in reserve as the random term. Here and subsequently, we visually inspected quantile–quantile plot of residuals for normality using the qqnorm function (base R). The AMF colonization model for native plants did not meet model assumptions due to large differences in variance within groups (i.e. high variance in uninvaded soils, progressively lower averages with less variation with more conditioning; see Supplementary Figure S2); therefore, we included a variance function that allowed for variance to vary based on the soil conditioning experimental groups for this model (as per Pinheiro and Bates 2006).

Soil-biota-feedback model post-hoc tests include a Tukey honestly significant difference test (Tukey HSD) to infer differences among groups within a significant fixed effect (i.e. soil conditioning, species, or soil conditioning × species), as well as a *t*-test to infer that treatment groups are statistically different from 0, indicating biologically different from the sterile conditions. The AMF colonization model post-hocs only include Tukey HSD for significant fixed effects.

To link AMF colonization and native soil-biota-feedback, we performed a linear mixed effects model focused on native soil-biota-feedback with AMF colonization, species, *Oncosiphon* soil conditioning, and the interactions (i.e. AMF colonization × species, AMF colonization × soil conditioning, AMF colonization × species × soil conditioning) as fixed effects, with soil core nested in reserve as the random term.

To assess if functional traits change in response to soil conditioning, we performed a linear mixed effects model focused on either SRL or SLA (i.e. two separate models) with soil conditioning, species, and the interaction with core nested in reserve as the random term for all species including *Oncosiphon*. To address how functional traits mediate responses to invader soil conditioning, we performed a linear mixed effects model focused on all species soil-biota-feedback (i.e. including *Oncosiphon*), with the fixed effects of soil conditioning, species, SLA, SRL, and all interactions (i.e. 4-way model) with soil core nested in reserve as the random effect.

All analyses except the AMF colonization response were run utilizing the ‘lme4’ package (Bates et al. 2015) in R (Version 4.3.3), while AMF colonization was assessed with ‘nlme’ package (Pinheiro and Bates 2006) with the ‘varIdent’ function to account for heteroscedasticity in the native AMF root colonization model (Pinheiro and Bates 2025). Model results were analysed with the ‘car’ package with type III sum of squares (Fox and Weisberg 2019), *post hoc*-tests were performed using the ‘emmeans’ package (Russell 2022) for Tukey HSD and the *t*-test function in base R for the secondary post-hoc tests.

## Results

### Oncosiphon response to own soil conditioning

**Figure 1:**
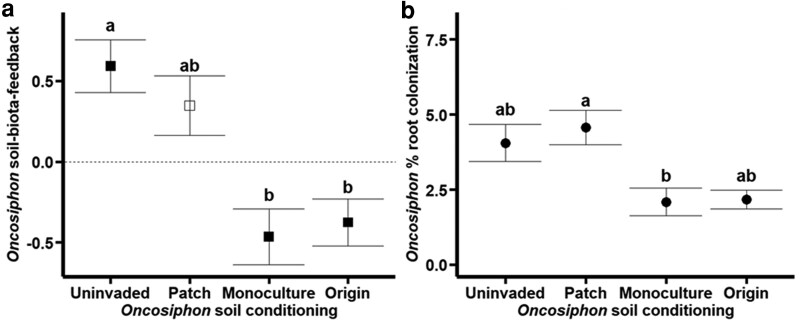
Oncosiphon response to Oncosiphon soil conditioning. a) Average Oncosiphon soil-biota-feedback (biomass grown in live inocula/biomass grown in sterile inocula) for each soil conditioning level, with standard error bars. Letters display the results of Tukey HSD. Filled points show the average soil-biota-feedback is significantly different from 0 (via *t*-test), indicating a significant difference in growth compared to sterilized soil. b) Average Oncosiphon percent root mycorrhizal colonization for each soil conditioning level with standard error bars. Letters display the results of Tukey HSD.

*Oncosiphon* growth decreased over the levels of soil conditioning such that in monoculture and origin inocula, *Oncosiphon* trended towards higher growth in sterile soil (Fig. 1a). This dynamic resulted in significant differences in *Oncosiphon* soil-biota-feedback across the sampled gradient (soil conditioning: *P* = 0.005, *F* = 5.17, DenDF = 31.9, see Table 1 for measurement averages). Specifically, *Oncosiphon* had greater growth in live inocula from uninvaded and patchy invaded inocula compared to sterile soil, resulting in similarly positive soil-biota-feedback (Tukey HSD *P* = 0.839). Meanwhile, *Oncosiphon* grown in live inocula from monocultures and the origin of invasion soil cores had lower growth than their respective sterilized soils, resulting in similarly negative soil-biota-feedback (Tukey HSD *P* = 0.981). *Oncosiphon* grown with inocula from uninvaded soils had higher soil-biota-feedback than monoculture and origin inocula (Tukey HSD: monoculture *P* = 0.012, origin *P* = 0.030). Additionally, *Oncosiphon* grown in uninvaded inocula derived detectable growth benefits from soil (*t*-test: *P* = 0.001), which were lost in patchily invaded inocula (*t*-test: *P* = 0.074). Individuals grown in monoculture and origin inocula had soil-biota-feedback significantly less than 0 (*t*-test: monoculture *P* = 0.013, origin *P* = 0.016), indicating plants grew more in sterile soil than in live soil.

**Table 1:** Average and standard error of responses to Oncosiphon soil conditioning.

Response variable	Treatment level	Species						
		*Oncosiphon*	*Amsinckia*	*Eschscholzia*	*Lasthenia*	*Layia*	*Lupinus*	*Nemophila*
Soil-biota-feedback	Uninvaded	0.59 ± 0.16	0.06 ± 0.17	−0.16 ± 0.24	0.70 ± 0.26	−0.11 ± 0.17	0.66 ± 0.17	0.57 ± 0.12
	Patch	0.35 ± 0.19	0.26 ± 0.16	0.24 ± 0.26	−0.15 ± 0.14	−0.70 ± 0.25	0.45 ± 0.28	0.40 ± 0.17
	Monoculture	−0.46 ± 0.17	−0.33 ± 0.18	−0.17 ± 0.23	0.08 ± 0.15	−0.23 ± 0.21	0.36 ± 0.14	−0.05 ± 0.13
	Origin	−0.38 ± 0.15	0.05 ± 0.15	0.38 ± 0.23	−0.04 ± 0.16	−0.66 ± 0.33	−0.20 ± 0.26	−0.09 ± 0.11
AMF colonization	Uninvaded	4.05 ± 0.62	31.33 ± 4.74	7.05 ± 1.85	29.18 ± 4.82	32.39 ± 3.42	36.56 ± 4.09	40.08 ± 3.60
	Patch	4.57 ± 0.58	41.52 ± 4.95	7.52 ± 1.30	15.10 ± 3.57	23.89 ± 4.38	31.56 ± 4.20	34.91 ± 3.57
	Monoculture	2.09 ± 0.46	13.52 ± 3.14	2.21 ± 0.39	3.91 ± 0.54	6.00 ± 1.54	4.47 ± 0.94	6.17 ± 0.73
	Origin	2.17 ± 0.31	11.59 ± 2.87	2.26 ± 0.70	2.81 ± 0.47	9.67 ± 2.69	7.10 ± 3.12	5.13 ± 0.69
Traits	SRL	224.5 ± 11.3	288.8 16.5	211.7 ± 10.6	357.2 ± 14.8	188.3 ± 7.8	126.8 ± 6.5	235.9 ± 8.4
	SLA	284.5 ± 14.5	322.0 ± 9.7	284.4 ± 13.3	391.7 ± 17.5	359.0 ± 15.3	300.9 ± 14.5	247.5 ± 7.4

The top section is soil-biota-feedback by soil conditioning, the middle section is percent mycorrhizal colonization by soil conditioning, and the bottom section is the average and standard error for traits (SLA, cm^2^/g and SRL, mg/m) independent of soil conditioning.

In contrast to the native plants, AMF colonization rates were generally low in *Oncosiphon*, with the highest overall percentage infected being ∼9% in the patchy inoculum. Despite the overall low colonization, *Oncosiphon* root mycorrhizal colonization varied across soil histories (soil conditioning: *P* = 0.014, *F* = 4.11, DenDF = 33.1, Fig. 1b), with colonization being significantly greater in patchy invaded inocula compared to monoculture inocula (Tukey HSD *P* = 0.035). *Oncosiphon* AMF colonization was similar between all other comparisons (Tukey HSD: uninvaded-patchy *P* = 0.939, uninvaded-monoculture *P* = 0.137, uninvaded-origin *P* = 0.218, patchy-origin *P* = 0.061, monoculture-origin *P* = 0.992).

### Native forb response to Oncosiphon soil conditioning:

**Figure 2:**
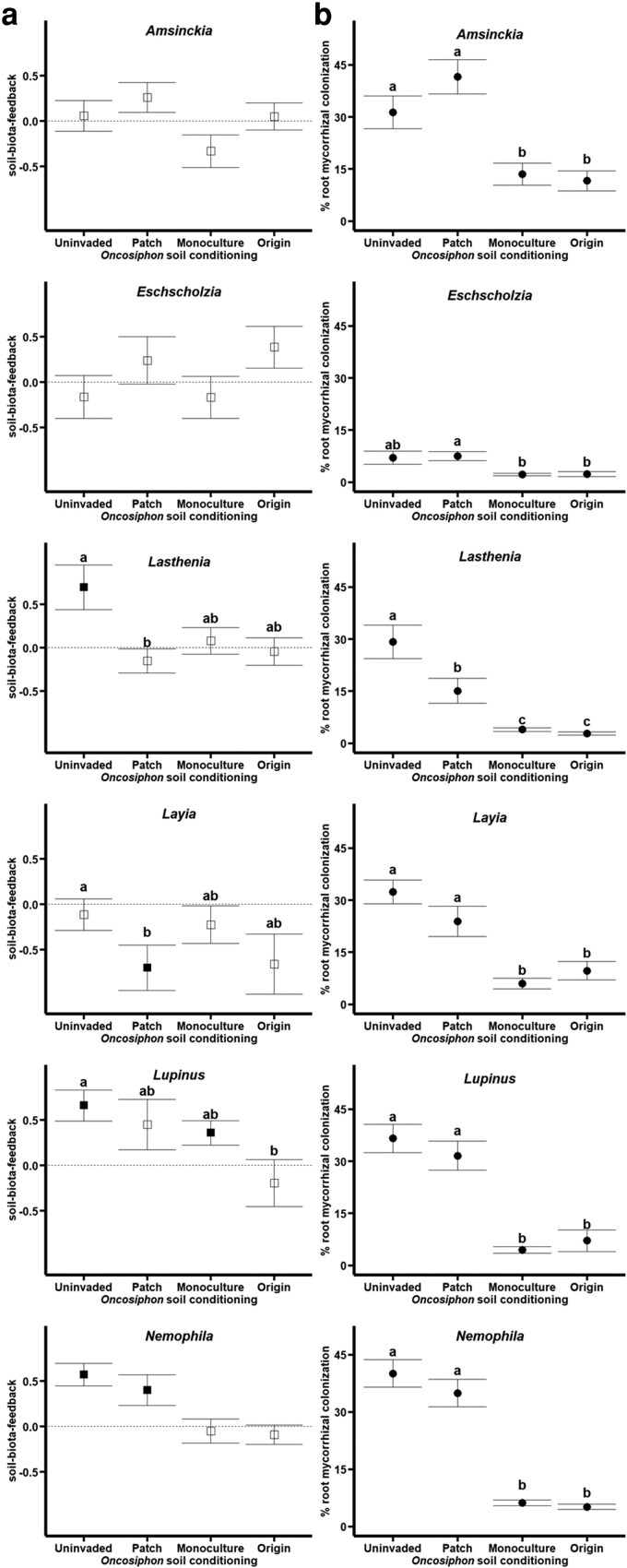
Native plant responses to Oncosiphon soil conditioning. a) Soil-biota-feedback (biomass grown in live inocula/biomass grown in sterile inocula) for native plants for each invader soil conditioning level with standard error bars. Filled points show the soil-biota-feedback is significantly different from 0 (via *t*-test), indicating a significant difference in growth compared to sterilized soil. Letters displayed are the results of Tukey HSD. b) Percent mycorrhizal colonization for each species across soil conditioning levels, with standard error bars. Letters indicate the results of Tukey HSD.

Native plant growth was not detectably influenced by the level of *Oncosiphon* soil conditioning alone (soil conditioning *P* = 0.277, *F* = 1.35, DenDF = 32.0); however, soil-biota-feedback differed between species (species *P* < 0.0001, *F* = 7.43, DenDF = 510.4) and this dynamic depended on the level of soil conditioning (species × soil conditioning *P* = 0.0004, *F* = 2.75, DenDF = 510.4, Fig. 2a). *Amsinckia* and *Eschscholzia* had minimal response to soil conditioning as soil-biota-feedback were close to neutral across all live sampled soil inocula. The growth benefit *Lasthenia* experienced with uninvaded inocula was lost at the next level of patchy soil conditioning (Tukey HSD *P* = 0.032), and it remained neutral for the other levels of soil conditioning. *Lupinus* experienced a gradual decrease in soil biota feedback with soil conditioning such that uninvaded inocula conferred a significantly higher soil-biota-feedback than origin inocula (Tukey HSD *P* = 0.045). Within the uninvaded soils, *Lasthenia*, *Lupinus*, and *Nemophila* grew more in live soil than sterile soil (*t*-test *P* = 0.011, 0.001, 0.0001, respectively), and *Nemophila* retained these growth benefits in patchily invaded soils (*t*-test *P* = 0.026). Meanwhile, *Lupinus* lost detectable growth benefits in patchily invaded soils (*t*-test *P* = 0.125), but did have detectably greater soil-biota-feedback in monoculture soils (*t*-test *P* = 0.015). The only native plant experiencing reduced growth in live soil inocula was *Layia* in the patchily invaded soil inocula (*t*-test *P* = 0.012).

Native root colonization rates significantly changed with *Oncosiphon* soil conditioning independent of plant species (soil conditioning: *P* < 0.0001, *F* = 17.19, DenDF = 32.0), where plants grown in uninvaded and patchy invaded inocula had similarly high AMF colonization (Tukey HSD *P* = 0.700) and plants grown in monoculture and origin inocula had similarly low AMF colonization (Tukey HSD *P* = 0.971). The native plant AMF colonization independent of species was greater in uninvaded and patchy inoculum than in monoculture and origin inoculum (Tukey HSD: uninvaded-monoculture *P* < 0.0001, uninvaded-origin *P* < 0.0001, patchy-monoculture *P* < 0.0001, patchy-origin *P* < 0.0001).

Species differed in their AMF colonization rates independent of soil conditioning (species: *P* < 0.0001, *F* = 16.44, DenDF = 460.0), where *Eschscholzia* had the lowest average root colonization compared to all other native species with an average colonization rate of 4.79 ± 0.66% (mean ± standard error; Tukey HSD *Amsinckia P* < 0.0001, *Lasthenia P* < 0.0001, *Layia P* < 0.0001, *Lupinus P* < 0.0001, *Nemophila P* < 0.0001). Additionally, *Lasthenia* had a lower average root colonization independent of soil conditioning than *Amsinckia* (Tukey HSD *P* < 0.0001), *Layia* (Tukey HSD *P* = 0.004), *Lupinus* (Tukey HSD *P* = 0.015), and *Nemophila* (Tukey HSD *P* = 0.001). Four species: *Amsinckia*, *Layia*, *Lupinus*, and *Nemophila*, had similar average root colonization rates. Native root colonization rate responses to *Oncosiphon* soil conditioning were mediated by species (soil conditioning × species: *P* = 0.043, *F* = 1.44, DenDF = 187.0), but the overall patterns were generally the same within species (Fig. 2b).

**Figure 3:**
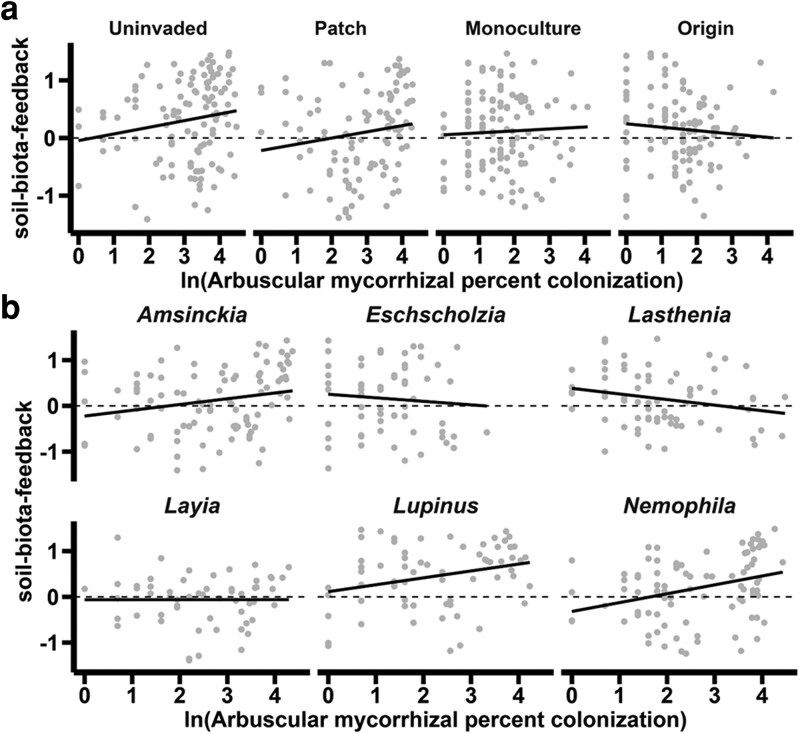
Relationships between soil-biota-feedback (biomass grown in live inocula/biomass grown in sterile inocula) and AMF root colonization rates. a) Relationship between natural log-transformed percent AMF colonization and soil-biota-feedback per soil conditioning level. The dashed line is at 0, which is statistically identical to sterile soil b) Species level relationships between natural log-transformed percent AMF colonization and soil-biota-feedback with a dashed line on 0 to indicate sterile values of soil-biota-feedback.

AMF root colonization was not, independently, related to soil-biota-feedback (AMF: *P* = 0.609, *F* = 0.26, DenDF = 459.55), but the relationship between AMF colonization and soil-biota-feedback was dependent on both soil conditioning (soil conditioning × AMF: *P* = 0.0006, *F* = 5.94, DenDF = 458.78, Fig. 3a) and species (species × AMF: *P* = 0.003, *F* = 3.68, DenDF = 450.44). Plants with higher AMF colonization derived more growth benefits in uninvaded soils, but the relationship became more neutral as *Oncosiphon* soil conditioning increased, eventually trending towards a negative association between root colonization and growth (Fig. 3a). Increased AMF colonization drove slightly higher soil-biota-feedback for *Amsinckia*, *Lupinus*, and *Nemophila* (Fig. 3b). Meanwhile, both *Eschscholzia* and *Lasthenia* had slightly reduced growth with higher AMF colonization, and *Layia* had a neutral relationship (Fig. 3b). Soil conditioning and species level differences were significant with consistent patterns described in previous analysis (soil conditioning: *P* = 0.003, *F* = 4.75, DenDF = 245.0; species: *P* = 0.0001, *F* = 5.14, DenDF = 448.2), and the relationship between AMF colonization, species, and soil history was not significant (species × soil history: *P* = 0.101, *F* = 1.50, DenDF = 446.4; AMF colonization × species × soil conditioning: *P* = 0.648, *F* = 0.83, DenDF = 451.6).

### Plant-trait ∼ soil-biota-feedback relationships:

Our assessment of *Oncosiphon* soil conditioning impacts on traits did not find significant differences across *Oncosiphon* invasion levels (soil conditioning: SRL *P* = 0.101, *F* = 2.25, DenDF = 34.2; SLA *P* = 0.264, *F* = 1.38, DenDF = 34.7; soil conditioning × species: SRL *P* = 0.548, *F* = 0.92, DenDF = 553.7; SLA *P* = 0.904, *F* = 0.595, DenDF = 557.9). However, species differed from each other for both SRL (species: *P* < 0.0001, *F* = 52.36, DenDF = 554.13, Supplementary Figure S3a) and SLA (species: *P* < 0.0001, *F* = 9.41, DenDF = 558.1, Supplementary Figure S2b). In comparing *Oncosiphon* to the native species, *Lupinus* had shorter, thicker roots (Tukey HSD *P* < 0.0001) while *Amsinckia* and *Lasthenia* had longer thinner roots than *Oncosiphon* (Tukey HSD: *Amsinckia P* = 0.002, *Lasthenia P* < 0.0001), but no other species had detectably different SRL (Tukey HSD: *Eschscholzia P* = 0.964, *Layia P* = 0.286, *Nemophila P* = 0.714). Meanwhile, for SLA, *Oncosiphon* had thicker, smaller leaves than *Lasthenia* (Tukey HSD *P* < 0.0001) and *Layia* (Tukey HSD *P* = 0.023), but SLA was similar between the other native plants and *Oncosiphon* (Tukey HSD: *Amsinckia P* = 0.404, *Eschscholzia P* = 0.994, *Lupinus P* = 0.994, *Nemophila P* = 0.334).

Within native species comparisons for SRL, *Lasthenia* had the longest, thinnest roots (Tukey HSD: *Amsinckia P* = 0.0008, *Eschscholzia P* < 0.0001, *Layia P* < 0.0001, *Lupinus P* < 0.0001, *Nemophila P* < 0.0001; Supplementary Figure S3a), while *Lupinus* had the shortest, thickest roots (Tukey HSD: *Amsinckia P* < 0.0001, *Eschscholzia P* < 0.0001, *Layia P* < 0.0001, *Nemophila P* < 0.0001). *Amsinckia* had longer, thinner roots than *Eschscholzia* (Tukey HSD *P* = 0.0001) and *Layia* (Tukey HSD *P* < 0.0001) but similar SRL as *Nemophila* (Tukey HSD *P* = 0.216). *Nemophila* had longer, thinner roots than *Layia* (Tukey HSD *P* = 0.007) and similar SRL as *Eschscholzia* (Tukey HSD: *P* = 0.211), while *Eschscholzia* and *Layia* had similar SRL (Tukey HSD *P* = 0.876, Supplementary Figure S2a). For SLA, *Lasthenia* had broader, thinner leaves than *Amsinckia*, *Eschscholzia*, *Lupinus*, and *Nemophila* (Tukey HSD: *Amsinckia P* = 0.002 *Eschscholzia P* < 0.0001, *Lupinus P* < 0.0001, *Nemophila P* < 0.0001) but similar SLA as *Layia* (Tukey HSD *P* = 0.725, Supplementary Figure S3b). Both *Amsinckia* and *Layia* had similarly broad thin leaves (Tukey HSD *P* = 0.366) with higher SLA than *Nemophila* (Tukey HSD: *P* = 0.0007, *P* < 0.0001). *Layia* had higher SLA than *Eschscholzia* (Tukey HSD *P* = 0.005) and *Lupinus* (*P* = 0.041), while *Amsinckia* had similarly thin leaves as both *Eschscholzia* (Tukey HSD *P* = 0.519) and *Lupinus* (Tukey HSD *P* = 0.910). Meanwhile, *Eschscholzia*, *Lupinus*, and *Nemophila* had similar SLA (Tukey HSD: *Eschscholzia-Lupinus P* = 0.995, *Eschscholzia-Nemophila P* = 0.381; *Lupinus-Nemophila P* = 0.091, Supplementary Figure S3b).

**Figure 4:**
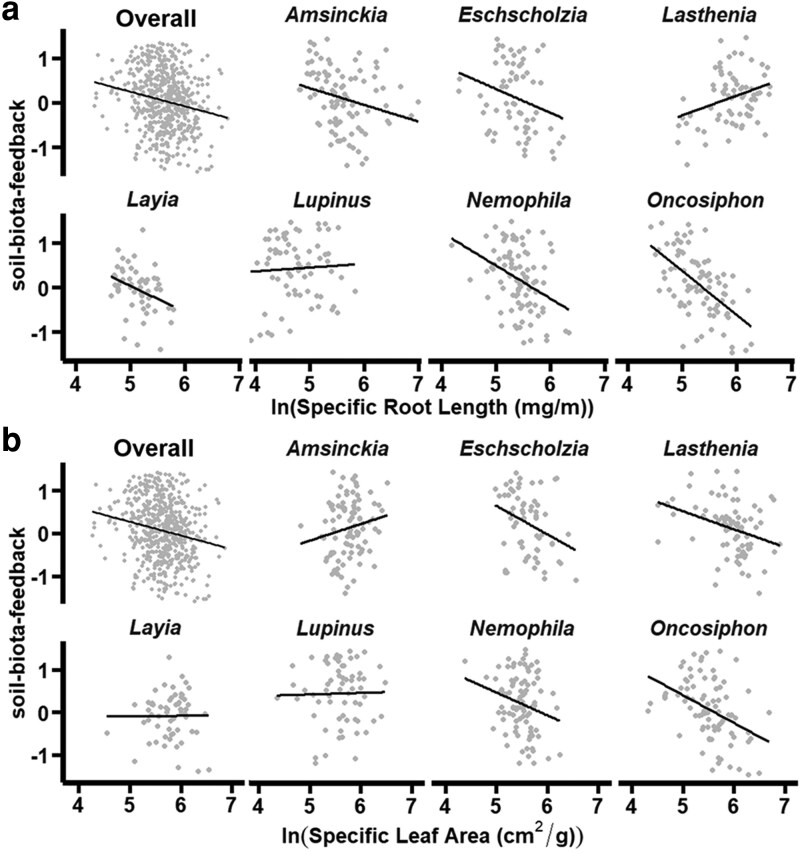
Estimated relationships between soil-biota-feedback (biomass grown in live inocula/biomass grown in sterile inocula) and functional traits, including SRL (mg/m) and SLA (cm^2^/g). a) Relationship between natural log-transformed percent AMF colonization and soil-biota-feedback pooled across soil conditioning level. b) Species-level relationships between AMF colonization and soil-biota-feedback.

In utilizing resource acquisition traits to mediate all investigated species responses to *Oncosiphon* soil conditioning, both SRL and SLA had a negative association with soil-biota-feedback overall (SRL: *P* = 0.022, *F* = 5.28, DenDF = 410.4. SLA: *P* = 0.029, *F* = 4.79, DenDF = 411.0, Fig. 4, see Supplementary Table S2 for full summary statistics); however, species mediated these trait-soil-biota-feedback relationships (SRL × species: *P* = 0.002, *F* = 3.51, DenDF = 418.7, Fig. 4a; SLA × species *P* = 0.002, *F* = 3.51, DenDF = 418.7, Fig. 4b). For *Amsinckia*, *Eschscholzia*, *Layia, Nemophila*, and *Oncosiphon*, individuals with longer thinner roots had lower soil-biota-feedback (Fig. 4a). The relationship between SRL and soil-biota-feedback was weak for *Lupinus* but positive for *Lasthenia* (Fig. 4a). Meanwhile, plants with larger thinner leaves (i.e. high SLA) had lower soil-biota-feedback for *Eschscholzia*, *Lasthenia*, *Nemophila*, and *Oncosiphon.* The association between SLA and soil-biota-feedback was weak for *Layia* and *Lupinus*, but higher SLA plants had more neutral trending towards positive soil-biota-feedback for *Amsinckia* (Fig. 4b).

**Figure 5:**
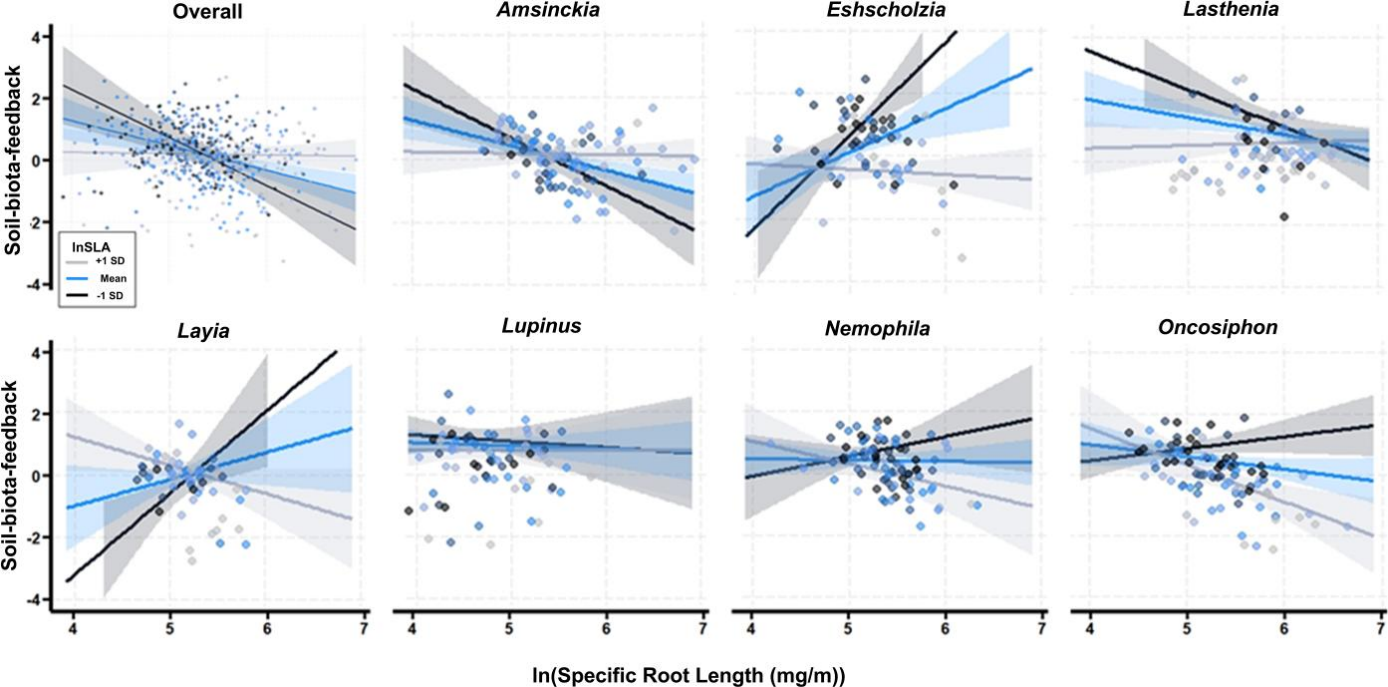
Interaction between SRL and SLA on the soil-biota-feedback. a) Overall interaction between SRL and SLA, where the blue indicates average SLA, black indicates SLA one standard deviation lower than the average, and grey indicates SLA one standard deviation higher than the average. Points are model residuals, as plotted by interact_plot(), and shaded bands represent ± standard error (estimated via 68% confidence interval) around model predictions. b) Species-specific interactions between SRL and SLA. Blue indicates the average SLA, black indicates the low estimate of SLA, and grey indicates the high estimate for SLA. Points are model residuals, as plotted by interact_plot().

Importantly, SLA mediated the relationship between SRL and soil-biota-feedback (SRL × SLA: *P* = 0.009, *F* = 6.97, DenDF = 410.53, Fig. 5, see Supplementary Table S2 for summary statistics), where overall, plants with lower SLA had a stronger negative relationship between SRL and soil-biota-feedback (i.e. low SRL higher soil-biota-feedback and high SRL lower soil-biota-feedback) and plants with higher SLA had a more neutral SRL-soil-biota-feedback relationship. This pattern of trait interaction was consistent for *Amsinckia* and *Lasthenia*, but the other species investigated demonstrated different patterns (SRL × SLA × species: *P* = 0.001, *F* = 3.81, DenDF = 418.48, Fig. 5). For *Eschscholzia*, *Layia*, *Nemophila*, and *Oncosiphon*, the relationship between SRL and soil-biota-feedback transitioned from positive to negative as SLA increased (black line to grey line in Fig. 5). Lastly, the slightly negative relationship between SRL and soil-biota-feedback was not mediated by SLA for *Lupinus.*

## Discussion

Our study demonstrates that *Oncosiphon* soil conditioning drives a dynamic negative response in most native species and highlights the importance of functional traits in shaping soil-biota-feedback responses. We found evidence for three key soil-biota-feedback processes occurring within the *Oncosiphon* invasion. (i) *Oncosiphon* itself derived growth benefits from soil prior to invasion, which were lost with invasion and soil-biota-feedback became negative at later stages of invasion. (ii) The soil-biota-feedback for most native species declined with *Oncosiphon* soil conditioning, often by early (i.e. patch inoculum) stages of invasion; however, AMF colonization rates for all native species declined significantly in later stages (i.e. monoculture inoculum), suggesting a potential threshold in fungal responses that lag behind lost plant growth benefits. Importantly, responses of native forbs varied widely and independent of soil conditioning, where certain species (*Amsinckia* and *Eschscholzia*) did not have a detectably different response to growing in live inocula, while others had a growth benefit (*Lasthenia*, *Lupinus*, and *Nemophila*) or a reduction in growth (*Layia*) from live inocula independent of *Oncosiphon* soil conditioning. (iii) Most native species exhibited both a do-it-yourself vs outsourcing tradeoff (i.e. higher SRL having lower soil-biota-feedback), with only half also exhibiting a growth-defense tradeoff (i.e. higher SLA having lower soil-biota-feedback). Additionally, we found evidence that SRL and SLA may work in tandem to mediate responses to soil biota, but that these responses differ by species. For example, for *Eschscholzia*, *Layia*, *Nemophila*, and *Oncosiphon* if an individual has either low SLA and low SRL or high SLA and high SRL they experience neutral or negative soil-biota-feedback, but having opposite strategies (e.g. low SRL and high SLA) will allow them to experience the benefits from soil biota (i.e. positive soil-biota-feedback). Together, our results highlight how the development of invader soil conditioning can reduce native forb performance and invader performance dynamically, and how trait coordination can mediate plant responses to soil conditioning.

Symbiotic mutualisms may provide a barrier to invasion success if a species requires obligate mutualisms; however, non-mycorrhizal or facultative mutualist invaders may not be strongly impacted by the availability of symbionts (Pringle et al. 2008). Here, the low AMF colonization rates within *Oncosiphon* roots suggest that AMF likely do not directly contribute to *Oncosiphon* performance. This response has been found in other invasive plants, including *Plantago virginica* (Luo et al. 2021), *Salsola tragus* (Hovland et al. 2019), and many species in the *Brassica* family (see Grove et al. 2017 for additional studies). Importantly, decreases in growth, driven by soil-biota in the late stages of invasion (i.e. monoculture and origin inocula), are likely insufficient to negatively impact the spread and dominance of this invader. *Oncosiphon* produces a large number of seeds per individual that can saturate the soil seedbank (Hedrick and McDonald 2020, Rodriguez et al. 2024), and it invades areas where the native seedbank has already been severely depleted (Cox and Allen 2008, Schwab et al. 2023). Moreover, waiting for negative soil-biota-feedback to decrease invader performance may allow for other invader-mediated legacies to develop that inhibit system recovery (Kelly et al. 2009, Kendig et al. 2021); therefore, management actions should be taken to constrain invader growth as early during an invasion as possible.

Plant associations with soil-biota (e.g. mycorrhizal dependency and sensitivity to pathogens) may mediate how strongly native plants are impacted by invader soil conditioning (Kelly et al. 2009, Vogelsang and Bever 2009, Kendig et al. 2021). In our study, the uninvaded inoculum provided a growth benefit to some native plants, but most native plants trended towards decreased soil-biota-feedback in inoculum with higher conditioning. By measuring AMF colonization rates, we were able to detect that native plant AMF colonization rates did not significantly decline until late stages of invasion (i.e. monoculture and origin inocula), despite early losses in growth benefits (i.e. patchy inoculum), suggesting a possible decoupling in fungal responses and lost growth benefits during plant invader soil conditioning. This decoupling of reductions in plant growth but delayed reductions in AMF colonization supports recent findings that invaders may first disrupt native performance by suppressing root nutrient acquisition vs disrupting mycorrhizal associations (Chen et al. 2022), as the performance of native plants decreased immediately with *Oncosiphon* presence, but the AMF colonization did not. This decoupling however did not translate to strong relationships between colonization and soil-biota-feedback, likely due to the variable responses across species. A species sensitivity to AMF can be mediated by their overall mycorrhizal dependency (i.e. obligate species are more sensitive) (Bunn et al. 2015). Microscopy approaches can quantify the degree of association between plants and soil symbionts, which can inform key tradeoffs in plant ecology, and pairing our microscopy with a sequencing approach could provide further insights into which specific subsets of AMF species are being affected along this invasion gradient.

Biological tradeoffs that mediate coordination between traits are important considerations when trying to link functional traits with the dynamics of invader soil conditioning. Current conceptual models predict that species with lower SRL will have more dependent associations with AMF (Bergmann et al. 2020), which is consistent with our findings as plants with low SRL had more positive soil-biota-feedback, and plants with high SRL had more negative soil-biota-feedback for all species except *Lasthenia*. In a recent review, Xi et al. (2021) demonstrated that higher SLA species experience more negative soil feedback likely due to increased sensitivity to pathogens, supporting that a growth-defense tradeoff mediates responses of native plants to soil-biota. With SLA, we observed a similar pattern demonstrating that higher SLA species experienced negative soil-biota-feedback, or a neutral relationship for most species, including *Oncosiphon*. However, *Amsinckia* showed the opposite pattern with higher SLA resulting in more positive soil-biota-feedback. The *Amsinckia* genus is one of the most common native plants in heavily invaded areas and has been described as ‘weedy’ (MacBride 1917, Montalvo et al. 2010) and *Amsinckia* is tolerant of other plant invader soil conditioning (Lankau and Strauss 2007), suggesting it may not be as susceptible to pathogens that drive the negative SLA-soil-biota-feedback relationship. Overall, the trait-soil-biota-feedback relationships we observed are consistent with expectations of growth-defense or outsourcing-do-it-yourself relationships found in other species.

The potential coordination between above and belowground traits is of considerable interest, as a global review recently identified four axes of trait coordination for above and belowground traits: conservation gradient (fast vs slow growth), collaboration gradient (do-it-yourself vs outsourcing), and two plant size gradients (plant height or rooting depth) (Weigelt et al. 2021). These axes are often explored independently, but our study suggests that strategies along the conservation gradient interact with the collaboration gradient to influence responses to soil-biota. A handful of studies that have investigated potential coordination between above and belowground traits have found whole plant strategies of high resource acquisition above and belowground (e.g. Candeias and Fraterrigo 2020, Ávila-Lovera et al. 2022, Mueller et al. 2024, Spitzer et al. 2025). In support of this coordination, *Amsinckia* and *Lasthenia* demonstrated that individuals with either low SLA and low SRL or high SLA and high SRL experienced positive or neutral soil-biota-feedback, respectively. However, we observed that this level of coordination was not effective for all species to resist *Oncosiphon* soil-biota feedback. For *Eschscholiza*, *Layia*, *Nemophila*, and even *Oncosiphon,* we found that plants with higher SRL and lower SLA had more positive soil-biota-feedback, thus for these species, exhibiting opposite strategies on the conservation and collaboration trait gradients allowed them to benefit from the live soil inocula despite invader soil conditioning. The relationship between plant traits and heterospecific feedback (i.e. soil-biota-feedback mediated by a different species) that is inherent in understanding invader soil conditioning is underexplored even in uninvaded plant communities, where the focus is on conspecific effects on soils (Kuťáková et al. 2018). Therefore, more studies like ours are needed that explore the responses of multiple native species to invader soil conditioning. Importantly, our data suggest that intraspecific variation in traits is important to consider when evaluating how traits mediate plant responses to invader soil-biota-feedback.

The legacies of invasion are complex, where invader-driven losses in AMF can have impacts years after invader removal (e.g. Lankau and Lankau 2014). The temporal dynamics and historical factors that affect soil should be included to isolate the effects of invaders and elucidate when invaders are drivers or passengers of change in communities. Here, using an example of a recent invader we found evidence of invader soil conditioning impacting native performance due to reduced AMF colonization, while concomitantly observing impacts on the invader itself. Our study came with a few practical constraints in the space-for-time substitution relying on the experience of land stewards with a qualitative estimate rather than an empirical estimate of invasion time, as well as the pooled sterilized controls, which limited the temporal resolution of the invader soil conditioning, and soil sterilization potentially increased nutrients so that plant reliance on symbionts is lessened. Additionally, we experienced ∼10% loss in data from unexpected mortality, which was especially pronounced within two species (*Layia* and *Lupinus*). While our pooling approach to address mortality did not strongly impact estimates of feedback (Brinkman et al. 2010), it may have influenced our ability to tease apart some soil history and trait interactions due to reduced sample sizes. Future studies should strive to balance testing more species while replicating to accommodate mortality. Further, soil and litter nutrient dynamics influence soil-biota-feedback (Bennett and Klironomos 2019), which our design does not incorporate. Nonetheless, the temporal development of these soil-biota-feedback mechanisms is nuanced as species may be able to buffer some of the impacts early during an invasion via their traits or collaborations with AMF. Trait-based approaches to predict native responses to invader soil conditioning might require integration of additional traits associated with differing types of symbioses, integration of nutrient dynamics, and more comprehensive species palettes with broader trait values to capture trait-soil-feedback relationships. Nevertheless, the impact of invasive species on soil and plant communities causes both short-term and long-term effects that depend on a variety of dynamic factors, such as invader residence time, the relationship between native plant functional traits and sensitivity to invader soil conditioning, and sensitivity of the soil community.

## Supplementary Material

plag005_Supplementary_Data

## Data Availability

Raw data and code are available as supporting information.
