# Supplementary material for: Invader soil conditioning impacts invader and native plant performance
Source: AoB Plants. 2026 Feb 3;18(1):plag005. doi: 10.1093/aobpla/plag005 (PMC12917917; doi:10.1093/aobpla/plag005)
Supplement: plag005_Supplementary_Data [file plag005_supplementary_data.zip › supplemental materials.pdf]

Supporting information for: **Invader soil conditioning impacts invader and native plant performance**

Appendix A) Soil handling techniques and preparation of inocula

Three 10cm diameter X 15cm deep cores were taken from each invasion level within each reserve. Soil cores were sieved with a sterilized (via 90% Ethanol spray) 2mm soil sieve to remove rocks and debris. We then measured 350ml of sieved soil for each live soil replicate and pooled the remaining soil replicates within each site and invasion level for the sterilized controls, thus we had four sterilized controls for each reserve. We pooled the 3 soil replicates for the sterilized controls to act as a biological control due to practical constraints of experiment size. Because we pooled replicates, the impacts on growth should be interpreted conservatively; however, the control is still an average of three plants in a greenhouse and most spatial structuring in PSF occurs with microbes (Reinhart & Rinella 2016, Rinella & Reinhart 2017), which are killed in the sterilization process. The sterilized inocula were steam-sterilized at 80˚C for 1 hour, rested for one hour, then steamed again for 1 hour. The sieved cores were stored in a refrigerator for no more than 72 hours before being used to inoculate pots in the greenhouse.

To create the bulk soil and to account for nutrient-based differences between reserves, we first collected background field soils from each reserve near the origin point of invasion. The background field soil was sieved through a 2mm sieve and mixed 1:1 with UC sand mix #3 (contents of soil mix in Table S1), then steam sterilized to 80˚C for 1 hour, rested for one hour, then sterilized again for 1 hour. We used a whole soil inoculation approach, where inoculum were added to pots at a ratio of 1:30 by volume of live soil to sterilized bulk soil from each reserve. The small amount of inoculant relative to bulk soil isolates the potential effects of biological drivers (Brinkman et al 2010).

References:

Brinkman PE, Van der Putten WH, Bakker E-J, Verhoeven KJF (2010) Plant-soil feedback: experimental approaches, statistical analyses and ecological interpretations. The Journal of ecology 98:1063–1073

Reinhart KO, Rinella MJ (2016) A common soil handling technique can generate incorrect estimates of soil biota effects on plants. The New phytologist 210:786–789

Rinella MJ, Reinhart KO (2017) Mixing soil samples across experimental units ignores uncertainty and generates incorrect estimates of soil biota effects on plants. The New Phytologist 216(1):15-17.

Supplementary Table 1) Contents of University of California Soil mix #3. Plaster sand and Peat moss are in percentage, and nutrients are in grams per cubic meter.

**Soil Content**	**Quantity**
Plaster Sand	0.57%
Peat Moss	0.43%
KNO3	86.70 g/m^3
Limestone Flour	520.19 g/m^3
Phosphate	433.50 g/m^3
Dolomite	1300.49g/m^3
Magnesium	24.28 g/m^3
Iron	45.08 g/m^3
Manganese	10.40 g/m^3
Zinc	17.34 g/m^3
Copper	38.15 g/m^3

Supplemental Table 2) Summary statistics of trait based model predicting all investigated species including *Oncosiphon* responses to *Oncosiphon* soil conditioning. SRL indicates specific root length (mg/m), SLA indicates specific leaf area (cm^2/g), and soil cond is shortened for conditioning where needed. DenDF is the denominator degrees of freedom, F indicates F ratio.

Factor	DenDF	F	p
SRL	410.4	5.28	0.022
SLA	411.0	4.79	0.029
Species	418.9	3.26	0.004
Soil Conditioning	410.8	0.48	0.699
SRL X SLA	410.5	6.97	0.009
SRL X species	418.7	3.51	0.002
SLA X Species	418.7	3.51	0.002
SRL X Soil conditioning	410.2	0.60	0.616
SLA X Soil. Conditioning	410.7	0.35	0.791
Species X Soil conditioning	418.5	1.07	0.376
SRL X SLA X Species	418.5	3.81	0.001
SRL X SLA X Soil cond.	410.2	0.45	0.716
SRL X Species X Soil cond	418.3	1.10	0.345
SLA X Species X Soil cond.	418.3	1.04	0.417
SRL X SLA X Species X Soil cond.	418.2	1.07	0.384

Supplemental Figures:

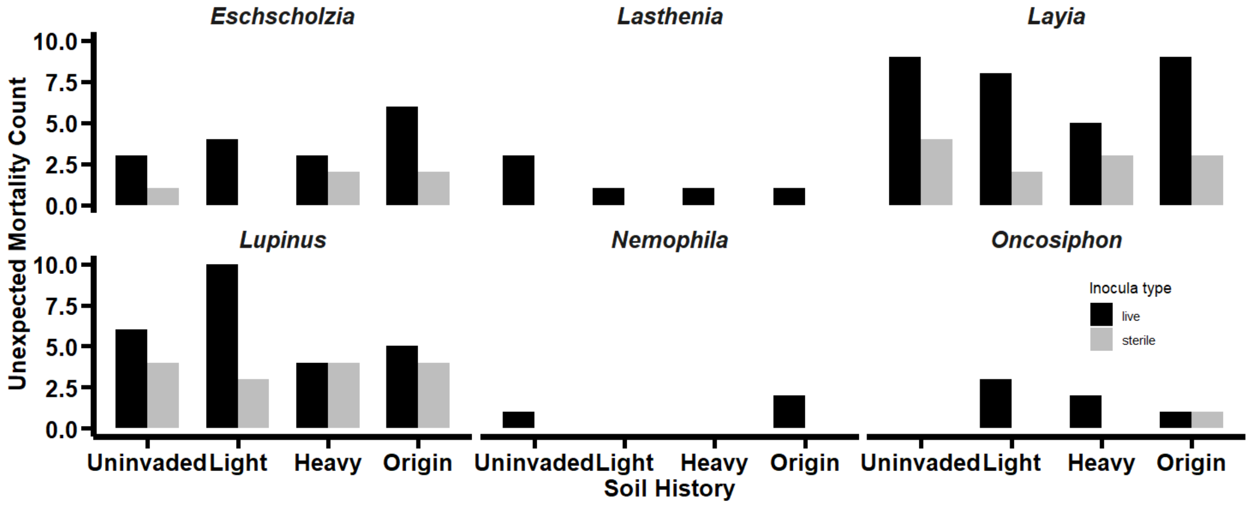

Supplemental Figure 1) Histogram of unexpected mortality within each soil history, for each species investigated except for *Amsinckia*, which did not experience unexpected mortality. The black bars represent unexpected mortality for replicates in living soil inocula, where the total N per treatment is 27. The grey bars represent replicates in sterilized soil inocula, where the total N per treatment is 9.

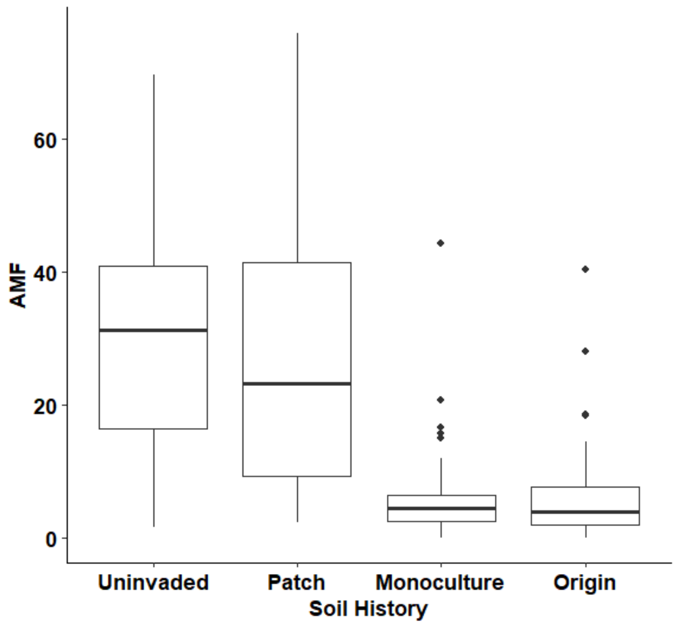

Supplemental Figure 2) Distribution of arbuscular mycorrhizal colonization (AMF) across soil invasion histories for all native species.

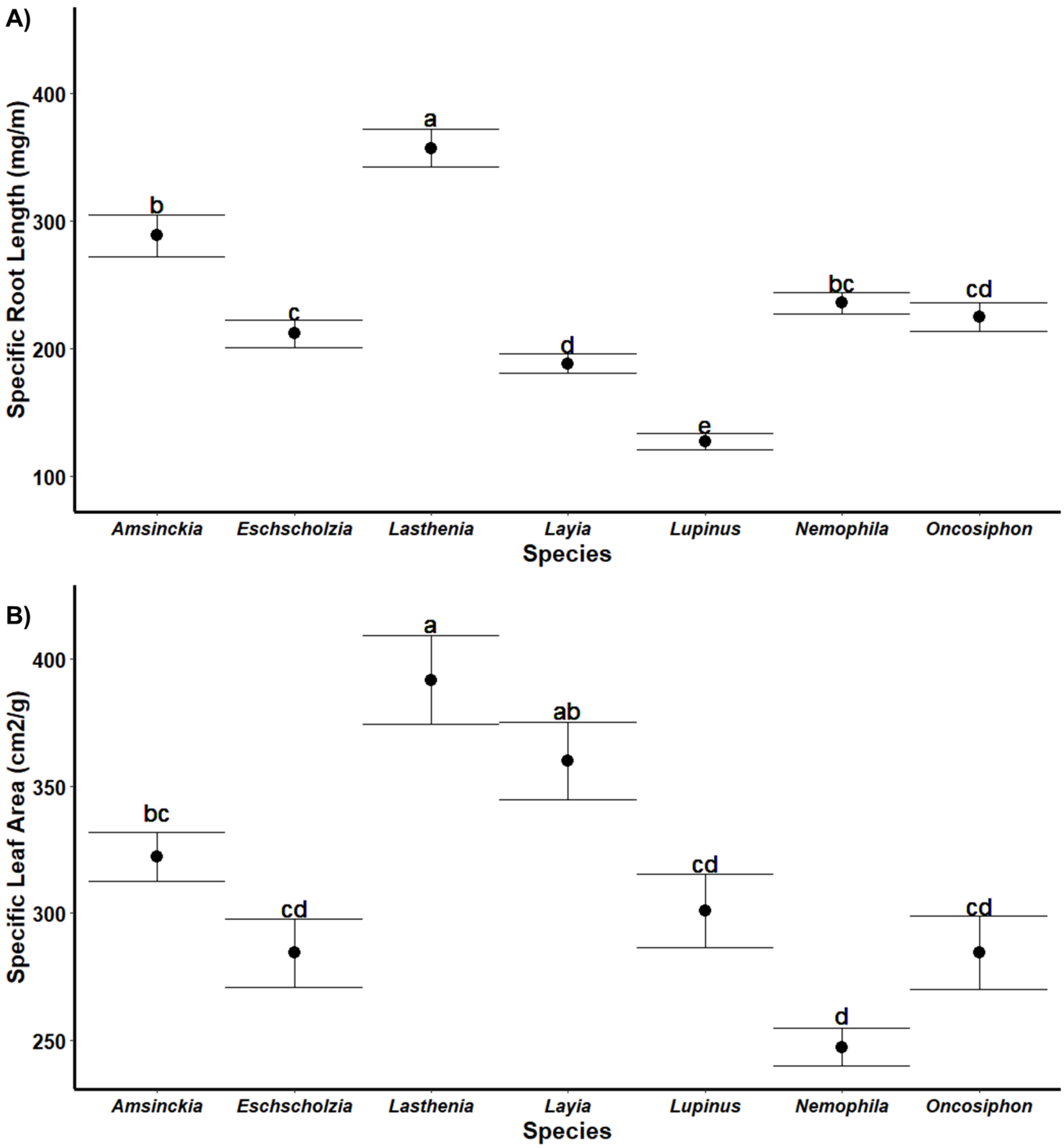

Supplemental Figure 3) Root and leaf functional traits for focal species pooled across soil conditioning treatments. A) Specific Root Length (SRL, mg/m) for all investigated species. B) Specific Leaf Area (SLA, cm^2/g) for all species investigated. Points show averages, error bars are standard error, and letters represent Tukey HSD significant comparison.
